# Supplementary material for: Application of a Novel Biosensor for Salivary Conductivity in Detecting Chronic Kidney Disease
Source: Biosensors (Basel). 2022 Mar 17;12(3):178. doi: 10.3390/bios12030178 (PMC8946539; doi:10.3390/bios12030178)
Supplement: Supplementary file 1 [file biosensors-12-00178-s001.zip › biosensors-1611523-supplementary.pdf]

Supplemental Materials

C-W Lin, Y-H Tsai and Y-P Lu, et al. Application of a Novel Biosensor for Salivary Conductivity in Detecting Chronic Kidney Disease

Content

Table S1. Co-linearity analysis of covariates put into the multivariate linear regression models.

Table S2-1. Characteristics of study populations without histories of diabetes and hypertension.

Table S2-2. Logistic regression analyses for the risk of chronic kidney disease in study populations without histories of diabetes and hypertension.

Table S3-1. Population characteristics in the propensity-score matched dataset.

Table S3-2. Logistic regression analyses for the risk of chronic kidney disease in the propensity-score matched dataset.

Figure S1. Conductance measurement of human saliva samples at different times.

Table S1. Co-linearity analysis of covariates put into the multivariate linear regression models.

	t	Sig.	Collinearity statistics	
			**Tolerance**	**VIF**
**Model 1**				
Constant	1.064	0.289		
Age	4.153	<0.001	0.745	1.342
Systolic blood pressure	0.929	0.354	0.880	1.137
BUN	-0.791	0.430	0.613	1.632
Fasting glucose	2.229	0.027	0.994	1.006
Creatinine	2.288	0.023	0.656	1.525
**Model 2**				
Constant	2.915	0.004		
Age	3.358	<0.001	0.679	1.473
Systolic blood pressure	1.007	0.315	0.884	1.132
BUN	-0.922	0.358	0.629	1.589
Fasting glucose	2.066	0.040	0.995	1.005
eGFR	-2.661	0.008	0.574	1.743

Abbreviations: BUN, blood urea nitrogen; eGFR, estimated glomerular filtration rate; VIF, the variance inflation factor

Table S2-1. Characteristics of study populations without histories of diabetes and hypertension.

	Low salivary conductivity group* (N=96)	High salivary conductivity group (N=40)	*p* value
**Salivary conductivity, ms/cm**	4.86±1.00	7.96±1.06	<0.01#
**Demographics**			
Age, years	58.48±14.83	70.93±12.22	<0.01#
Gender (male), n (%)	31 (32.3)	18 (45.0)	0.16
Body weight, kg	60.07±11.15	62.88±11.70	0.70
Body height, cm	159.55±7.79	160.26±8.26	0.63
Body mass index, kg/m2	24.31±3.38	24.43±3.76	0.85
Systolic blood pressure, mmHg	125.23±18.62	131.98±20.13	0.06
Diastolic blood pressure, mmHg	74.53±10.68	74.83±11.32	0.89
**Comorbid conditions, n (%)@**			
Chronic kidney disease	1 (1.0)	2 (5.1)	0.14
Ischemic heart disease/ Stroke	3 (3.1)	3 (7.7)	0.24
Dyslipidemia	7 (7.3)	1 (2.5)	0.28
Gout	2 (2.1)	2 (5.1)	0.34
Chronic liver disease	8 (8.3)	3 (7.7)	0.90
**Laboratory parameters**			
BUN, mg/dL	13.84±4.25	17.39±5.72	<0.01#
Creatinine, mg/dL	0.75±0.22	0.91±0.34	<0.01#
eGFR, mL/min/1.73 m2	93.66±19.81	77.98±21.81	<0.01#
Serum osmolality, mOsm/kgH2O	286.33±4.55	289.15±5.31	<0.01#
Fasting glucose, mg/dL	98.13±27.48	102.58±13.28	0.33
Hemoglobin A1c, %	5.75±1.06	5.71±0.41	0.81
ALT, U/L	22.05±21.58	22.08±10.66	0.58
Triglyceride, mg/dL	100.86±59.54	126.10±119.32	0.21
Total cholesterol, mg/dL	199.81±31.94	204.05±43.63	0.53
LDL-C, mg/dL	122.56±27.25	124.68±35.57	0.71
HDL-C, mg/dL	57.02±13.34	55.10±14.15	0.45

Values are expressed as mean ± standard deviation or number (percentage).

* Study populations were stratified into low and high groups according to the cutoff value of salivary conductivity (6.59 ms/m).

@ The information of comorbid conditions was obtained by questionnaires.

# indicates *p* value < 0.05.

Table S2-2. Logistic regression analyses for the risk of chronic kidney disease in study populations without histories of diabetes and hypertension.

	Odds ratio (95% CI)		
	Univariate	Multivariate	
		Model 1	Model 2
**Salivary conductivity***			
**High**	5.75# (1.62-20.39)	5.12# (1.39-18.86)	4.71# (1.24-17.84)
**Low**	(Ref)	(Ref)	(Ref)

Abbreviations: CI, confidence interval; Ref, reference
* Salivary conductivity here is a dichotomous variable by the cutoff value of 6.59 ms/m.
Model 1: adjusted for age, and gender
Model 2: adjusted for age, gender, and history of dyslipidemia
# indicates p value < 0.05.

Table S3-1. Population characteristics in the propensity-score matched dataset.

	**Low salivary conductivity group* (N=74)**	**High salivary conductivity group (N=74)**	***p* value**
**Salivary conductivity, ms/cm**	4.93±1.02	7.94±1.02	<0.01#
**Demographics**			
Age, years	67.61±9.36	69.92±10.93	0.17
Gender (male), n (%)	26 (35.1)	29 (39.2)	0.61
Body weight, kg	63.74±11.44	63.35±11.21	0.83
Body height, cm	158.05±8.14	158.76±8.24	0.60
Body mass index, kg/m2	25.44±3.50	25.07±3.40	0.51
Systolic blood pressure, mmHg	134.68±20.23	135.68±21.91	0.77
Diastolic blood pressure, mmHg	75.26±12.28	76.14±12.16	0.66
**Comorbid conditions, n (%)@**			
Diabetes mellitus	11 (14.9)	15 (20.3)	0.39
Hypertension	33 (44.6)	26 (35.1)	0.24
Chronic kidney disease	0 (0)	2 (2.7)	0.15
Ischemic heart disease/ Stroke	5 (6.8)	9 (12.3)	0.25
Dyslipidemia	18 (24.3)	10 (13.7)	0.09
Gout	4 (5.4)	5 (6.8)	0.73
Chronic liver disease	12 (16.2)	8 (11.0)	0.35
**Laboratory parameters**			
BUN, mg/dL	14.85±4.94	16.97±5.78	0.02
Creatinine, mg/dL	0.76±0.17	0.90±0.30	<0.01#
eGFR, mL/min/1.73 m2	88.46±19.79	77.17±21.27	<0.01#
Serum osmolality, mOsm/kgH2O	288.35±5.07	288.69±6.15	0.72
Fasting glucose, mg/dL	102.81±22.90	108.16±20.34	0.14
Hemoglobin A1c, %	5.94±0.85	5.97±0.83	0.88
ALT, U/L	21.39±9.80	20.62±9.74	0.63
Triglyceride, mg/dL	120.77±71.26	123.95±92.62	0.82
Total cholesterol, mg/dL	197.72±39.20	200.15±45.37	0.73
LDL-C, mg/dL	118.38±33.88	122.05±38.65	0.54
HDL-C, mg/dL	55.01±13.39	53.84±13.89	0.60

Values are expressed as mean ± standard deviation or number (percentage).
* Study populations were stratified into low and high groups according to the cutoff value of salivary conductivity (6.59 ms/m).
@ The information of comorbid conditions was obtained by questionnaires.
# indicates *p* value < 0.05.

Table S3-2. Logistic regression analyses for the risk of chronic kidney disease in the propensity-score matched dataset.

	Odds ratio (95% CI)		
	Univariate	Multivariate	
		Model 1	Model 2
**Salivary conductivity***			
**High**	5.52# (1.52-20.13)	4.63# (1.05-21.10)	4.47# (1.01-20.78)
**Low**	(Ref)	(Ref)	(Ref)

Abbreviations: CI, confidence interval; Ref, reference

* Salivary conductivity here is a dichotomous variable by the cutoff value of 6.59 ms/m.

Model 1: adjusted for age, and gender

Model 2: adjusted for age, gender, and histories of diabetes, hypertension, or dyslipidemia

# indicates p value < 0.05.

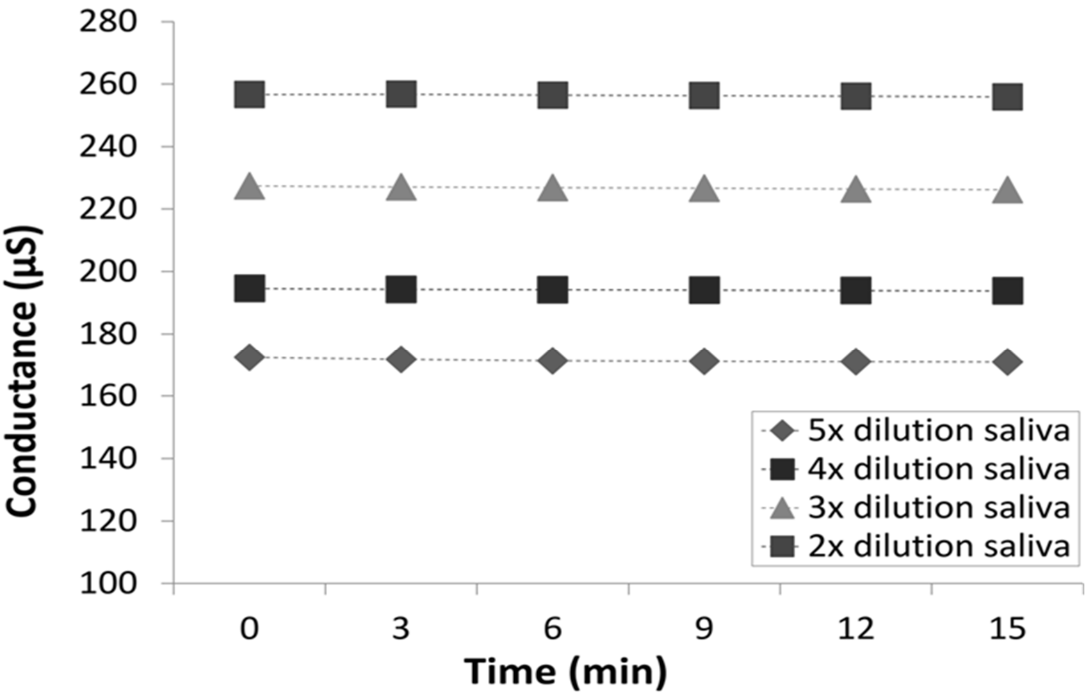

Figure S1 Conductance measurement of human saliva samples at different times.
